# A trajectory-based loss function to learn missing terms in bifurcating dynamical systems

**DOI:** 10.1038/s41598-021-99609-x

**Published:** 2021-10-14

**Authors:** Rahel Vortmeyer-Kley, Pascal Nieters, Gordon Pipa

**Affiliations:** Institute of Cognitive Science, Osnabrück University, Wachsbleiche 27, 49090 Osnabrück, Germany

**Keywords:** Nonlinear phenomena, Computational science

## Abstract

Missing terms in dynamical systems are a challenging problem for modeling. Recent developments in the combination of machine learning and dynamical system theory open possibilities for a solution. We show how physics-informed differential equations and machine learning—combined in the Universal Differential Equation (UDE) framework by Rackauckas et al.—can be modified to discover missing terms in systems that undergo sudden fundamental changes in their dynamical behavior called bifurcations. With this we enable the application of the UDE approach to a wider class of problems which are common in many real world applications. The choice of the loss function, which compares the training data trajectory in state space and the current estimated solution trajectory of the UDE to optimize the solution, plays a crucial role within this approach. The Mean Square Error as loss function contains the risk of a reconstruction which completely misses the dynamical behavior of the training data. By contrast, our suggested trajectory-based loss function which optimizes two largely independent components, the length and angle of state space vectors of the training data, performs reliable well in examples of systems from neuroscience, chemistry and biology showing Saddle-Node, Pitchfork, Hopf and Period-doubling bifurcations.

## Introduction

Interacting oceanic flows governing our climate, increasing ${\text {CO}}_2$ levels causing global warming, competing species for coexistence, dominance or extinction—we live in a changing world. Understanding the impact of these often slow and gradual changes is important. But sometimes, a system can undergo a sudden fundamental change and end up in a totally different dynamical behavior. These points are sometimes called tipping points or regime shifts. Since the middle of the last century, the theory of dynamical systems has been applied to characterize the behavior of changing systems and real world phenomena in particular^1–5^ and examples therein.

The dynamics of the system can be described by the behavior of trajectories in a space defined by the state variables of the system. This state space contains repelling, attracting and separating structures—namely stable and unstable manifolds, and separatrices, and different types of attractors and coherent structures—that can act as organizing structures by governing the dynamical behavior of trajectories^6,7^.

When modeling real world systems in simple equations we still face difficulties because our knowledge or understanding of the underlying processes is limited (e.g.^8–10^). Here machine learning can help discover missing knowledge about the dynamical relationships between state variables from observed data by statistical inference. For example, Universal Differential Equations (UDE)^11^ are a recently proposed method to learn dynamical systems from data with machine learning and can be combined with the Sparse Identification of Nonlinear Dynamics (SInDy) algorithm^12^ to estimate an algebraic form of the dynamical system from data (see the next section for details). We show how this approach can be applied to learn missing terms in systems that can undergo sudden fundamental changes in their dynamics called bifurcation.

The goal of this work is to investigate the role of the loss function used to compare the learned dynamics and the training data given as time series data in learning a UDE. In particular, we propose a new loss function for optimization that compares angle and length of vectors in state space independently. Usually, the mean-squared error is used to compare time series for each variable in the system independently. Our idea is that this new Length Difference and Angle Difference (LDA) loss is more reliable when learning a UDE in many bifurcating systems and therefore is better suited to find missing terms in bifurcating systems. The examples we use for this comparison cover on the one hand a broad range of different bifurcation types (Saddle-Node, Pitchfork, Hopf and Period-doubling bifurcation) and on the other hand show the importance of bifurcations in various fields of research (e.g. neuroscience, biology, chemistry). Firstly, we set up a statistical comparison of how well the two loss functions perform in each of these systems in two different parameter regimes representing two different dynamics respectively. We then narrow this comparison by focusing on nested intervals around the bifurcation parameter as well as different trajectory starting points in state space. Finally, we show by way of example that an algebraic reconstruction of the missing terms using SInDy is possible if a UDE was trained successfully and discuss our results.

### Universal differential equations (UDE) and sparse identification of nonlinear dynamics (SInDy)

**Figure 1:**
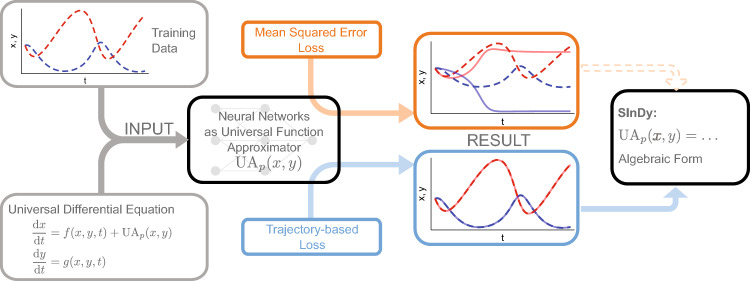
Schematic sketch of the concept of UDE^11^. Depending on a successful approximation of the training data, the application of SInDy^12^ is possible. The time series qualitatively correspond to the Selkov model^15^ in Table 2 second row. This figure is plotted using Inkscape (Version 1.0.1, https://inkscape.org) and Julia package Plots (Version v1.16.6, https://github.com/JuliaPlots/Plots.jl).

In this work, we investigate a complete, data-driven pipeline to discover the equations that describe a dynamical system in the context of bifurcating systems. The approach follows recently published work by *Rackauckas et al.* and combines the estimation of a UDE^11^ from data and the subsequent identification of algebraic terms from the UDE using SInDy^12^. We have illustrated the main steps involved in Fig. 1, starting with time series measurements from the system in question which will be used as training data.

Firstly, a Universal Differential Equation generally describes the dynamics of a system as ${\dot{\mathbf{x }}} = f(\mathbf{x }, t, {\text {UA}}_p(\mathbf{x }, t))$ where the function ${\text {UA}}_p$ is a parameterized and differentiable universal function approximator. For example, a partially known two dimensional system with variables *x* and *y* may be described as:

1 $$\begin{aligned} \begin{aligned} \frac{{\text {d}}x}{{\text {d}}t}&=f(x,y,t)+{\text {UA}}_p(x,y) \\ \frac{{\text {d}}y}{{\text {d}}t}&=g(x,y,t) \end{aligned} \end{aligned}$$

where functions *f* and *g* describe the known dynamics for *x* and *y* respectively, but we want to estimate an additional additive term in the dynamics of *x*. Given an initial $x(t_0)$ and $y(t_0)$ as well as initial parameters $p_{\text {init}}$ the UDE can numerically be solved and trajectories ${\hat{x}}(t)$ and ${\hat{y}}(t)$ are estimated. Using a loss function, the Mean Squared Error loss (cf. next section) for each variable of a system as in the original work, the estimated trajectories based on the UDE can be compared against the actual system measured at sample points $t_i$. The entire program, from the definition of the unknown function ${\text {UA}}_p$ and the UDE through to the numerical solution of the system and the calculation of the loss, can be differentiated with respect to parameters *p* using automatic differentiation in the Julia programming language^13,14^. This enables the optimization of these parameters by gradient descent on the loss function, and thereby allows a user to find a UDE with parameterized function ${\text {UA}}_{p~{\text {optimal}}}$ that approximates the measured time series of the original system well.

Using differentiable models in machine learning problems is a generalized view of highly successful deep and artificial neural network (ANN) models^16^ that have previously been used to solve differential equations^17,18^. Physics-informed neural networks, for example, aim to combine advantages of data-driven machine learning with knowledge about underlying physical laws in the training process to reconstruct dynamics of a system^19–21^. In this paper, we use ANNs as the parameterized universal function approximater^22,23^ ${\text {UA}}_p$. The disadvantage is that neural networks are black boxes and do not allow us to learn about systematic relationships between the variables of the dynamical systems that may be the drivers of underlying dynamics.

Therefore, we also use the SInDy algorithm^12^ to identify algebraic terms that can replace the neural network black box. In SInDy, finding the algebraic form of a differential equation is formulated as a linear regression problem. A matrix of non-linear functions $\Theta$ applied to the state vector ${\mathbf{x }}$ of the system multiplied by a matrix of sparse coefficient vectors $\Xi$ recovers the standard form of many non-linear dynamical systems: ${\dot{\mathbf{x }}} = f(\mathbf{x }) = \Theta (\mathbf{x }) \Xi$ (see the original paper^12^ for examples). If measurements for both $\mathbf{x }$ and ${\dot{\mathbf{x }}}$ are available at several sample points in time we can write ${\dot{X}} = \Theta (X) \Xi + \eta Z$ where *X* and ${\dot{X}}$ have rows for each sample point, $\Theta (X)$ is a design matrix of non-linear functions applied to the data, and $\eta Z$ is independent and identically distributed Gaussian noise with magnitude $\eta$. This is a standard linear regression problem in multiple variables^24^ where finding the sparse coefficient vectors $\Xi = [{\varvec{\xi }}_\mathbf{1 }, \ldots , {\varvec{\xi }}_\mathbf{n }]$ for a system with *n* state variables by using for example the LASSO algorithm^25^ can find those non-linear functions in $\mathbf{x }$ in the design matrix that best explain the data.

Often, data for $\mathbf{x }$ is not abundant and ${\dot{\mathbf{x }}}$ is not immediately available. In these cases, we can still estimate the UDE that fits the data. Given a UDE, it is easy to calculate ${\dot{\mathbf{x }}}$ from any given $\mathbf{x }$ and SInDy can be applied. Doing so will find functions in $\mathbf{x }$ that are much easier to understand and analyze than a neural network and we have successfully removed the black box from the UDE. Furthermore, if partial knowledge about the system is included in the UDE, we can still use SInDy to find algebraic terms for the contribution by solving the regression problem ${\text {UA}}_{p~\text {optimal}}(x) = \Theta (x)\Xi + \eta Z$ in isolation and complete the algebraic description of the dynamical system.

## Results

### The loss function and the trajectories

Generally speaking, the objective function or loss function in an optimization problem must be chosen in accordance with the goal of the optimized model. In neural network regression models, which we use here to find an approximation to missing terms in the UDE, this is typically done by minimization of the Mean Squared Error (MSE) loss function^26^. However, more specialized practitioners of model optimization have sometimes found that particular choices of one subtly different loss function over another can significantly alter the quality of the model fit, for example in financial modeling of option pricing^27^.

**Figure 2:**
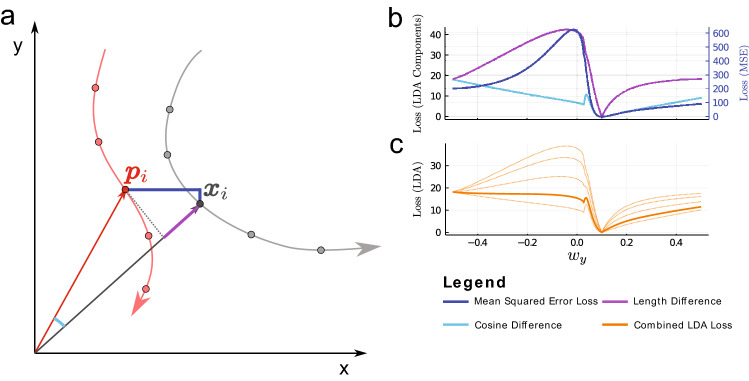
Schematic sketch of the concept of MSE and LDA loss function. (**a**) The UDE estimates trajectories $P=(\mathbf{p }_1, \mathbf{p }_2,\ldots ,\mathbf{p }_n)$ (red) in state space, the training data is $X=(\mathbf{x }_1,\mathbf{x }_2,\ldots ,\mathbf{x }_n)$ (gray). The MSE measures relative difference in each dimension of the sample point independently (blue lines), whereas the LDA measures the difference in angle (cyan) and difference in vector length (purple). (**b**) Comparing trajectories for the Selkov model^15^ (cf. Table 2 second row) based on MSE (blue), angular difference (cyan) via the cosine similarity and length difference (purple) all show a maximum that splits the loss landscape into two regions, one where the correct minimum can be reached and one where it cannot. Trajectories are compared based on a simple regression model in which only the weight for $y: w_y$ in the Selkov model is unknown and gives the *x*-axis here. (**c**) A weighted sum of the angular and length difference gives the LDA loss. Different sums are shown, with $k_1 = 0.25$ (length) and $k_2 = 0.75$ (angle) marked as the thick orange line that gives a good compromise between the two components. This figure is plotted using Inkscape (Version 1.0.1, https://inkscape.org) and Julia package Plots (Version v1.16.6, https://github.com/JuliaPlots/Plots.jl).

In the UDE approach, the MSE is used to compare observed dynamics to the estimated dynamics of the UDE by treating each component of the *i*-th sampled state space vector $\mathbf{x }_i$ (observed) and $\mathbf{p }_i$ (estimated) independently:

2 $$\begin{aligned} L_{\text {MSE}}= & {} \sum _{i=1}^{n} \sum _{j=1}^{m}(\mathbf{x }_i^j-\mathbf{p }_i^j)^2, \end{aligned}$$

where $\mathbf{x }_i^j$ resp. $\mathbf{p }_i^j$ is the j-th component in *m* dimensional state space, and the i-th point on a trajectory of length *n* (cf. blue difference vectors in Fig. 2a). This can work well in practice in many different UDE problems^11,28^. However, when trying to find a missing term in a potentially bifurcating system, this particular choice of loss function can lead to unsatisfactory results.

For example, if the data generating system can undergo a Hopf bifurcation from a stable state solution to an oscillatory solution, a UDE trained with an MSE loss often does not achieve dynamics in the correct regime and does not produce dynamics that fit the original system’s structure of nullclines and attractors (see animated state space portrait example of the oscillatory state of the Selkov model^15^ in the Supplementary video material S1 panel c).

How can the optimization fail so spectacularly? If we simplify the problem and replace the neural network by a simple linear regression with three parameters, one for each dimension of the state space and a bias parameter, we can visualize the loss function across any axis. If bias and weight for state variable *x* from the example in Fig. 1 (cf. Selkov model in Table 2 second row) are chosen correctly as 0, the correct solution for the weight for *y* is 0.1 in this case. Indeed, the MSE loss has its minimum at the correct value (Fig. 2b blue line), but it also has a significant peak just below 0. For values smaller than 0, the gradient-descent based optimization of the UDE runs into an alternative local minimum. This alternative local minimum acts like a “black hole of loss” in the loss landscape and cannot be escaped. It becomes impossible to find the true solution. Thus, the quality of the approximation is entirely dependent on initial conditions (see results below as well as the animated state space portrait in the Supplementary video material S1 panel c).

In the setup using a neural network, we also observe the phenomenon that trajectories of the estimated and real system must first diverge so that the correct dynamical regime can be reached. While the loss landscape over many ANN parameters is not easily visualized, it can still contain local minima that represent alternative stable solutions and act as “black holes of loss”.

We realized that this problem is unlikely to be solved by a single loss function and were inspired by the view of the dynamics as a trajectory of state space vectors. These can also be described by their angle and length always having the origin in state space as their common reference.

We construct the Length Difference and Angle Difference Loss (LDA):

3 $$\begin{aligned} L_{LDA}= & {} \sum _{i=1}^{n}\left( k_1 \cdot \underbrace{\frac{\sqrt{ (\left| \mathbf{x }_i\right| - \left| \mathbf{p }_i \right| )^2}}{\left| \mathbf{x }_i\right| + \left| \mathbf{p }_i \right| }}_{\text {length~difference}}+k_2 \cdot \underbrace{\frac{1}{2} \cdot \left( 1- \frac{\mathbf{x }_i \cdot \mathbf{p }_i}{\left| \mathbf{x }_i\right| \cdot \left| \mathbf{p }_i \right| }\right) }_{\text {angle~difference}}\right) , \end{aligned}$$

where the first component describes the normalized length difference between the position vectors $\mathbf{x }_i$ and $\mathbf{p }_i$ of the i-th point on a trajectory of length *n* and the second component the normalized difference between the angles between the two position vectors (namely a normalized cosine similarity, cf. Fig. 2a).

Each component of LDA individually also has false local minima (Fig. 2b purple and cyan), but, crucially at different points. However, the global minimum is at the same location in both functions. The ratio of these two components can be weighted with the hyperparameter $k_1$ and $k_2$ (Fig. 2c) chosen for each problem. In most cases $k_1$ and $k_2$ are chosen equal to 0.5 except for the Selkov model, where $k_1$ equals 0.2 and $k_2$ equals 0.8. Exceptions to these values are indicated in the text.

This constructed loss function can be used to train the UDE successfully and is less dependent on the initial ANN parameter guess. The UDE now reconstructs the state space portrait of the original describing differential equation faithfully (see the animated state space portrait in the Supplementary video material S1 panel a).

In the simplified example, the combined LDA loss cannot fully eliminate a second erroneous local minimum using a simple weighting of each component. However, when using a neural network as the model, the parameter space has much larger dimensionality. We hypothesized that the basin of attraction for a correct minimum is much larger when using the LDA loss with appropriate weight parameters. This should result in a much higher chance to find a UDE that fits the data given random initial parameters than the MSE loss. Furthermore, we expected this effect to also depend on the bifurcation itself. In the following we tested both hypotheses extensively in computational experiments on four example systems with bifurcations.

### The loss functions and different bifurcating systems

What happens if these loss functions are applied to different bifurcating systems from neuroscience, biology and chemistry? We considered the following four systems as examples of four different types of bifurcation:

- Saddle-Node bifurcation: The FitzHugh–Nagumo model^29,30^ describes resting and excited states of neurons. Specific parametrizations of the system can lead to a bistable behavior (“excitable”) with three fixed points where two are stable and one unstable (saddle). When the bifurcation parameter changes, the left stable fixed point and the saddle merge and disappear in a Saddle-Node bifurcation; the right stable fixed point remains and the system is monostable. (cf. Table 1)

**Table 1:** Overview of the models used with changing number of fixed points: FitzHugh–Nagumo model and Gardner model. First column: UDE for the systems in a general *x* and *y* notation with ${\text {UA}}_p(x,y)$ as machine learnable blank; second column: example of a trajectory’s longterm behavior trained with LDA (blue) in state space in comparison to the true solution’s trajectory (black).

	UDE	State space	Time series	SInDy estimate	
Monostable FitzHugh–Nagumo	$$\begin{array} {r@{}l@{}}\frac{\text {d}x}{{\text {d}}t} &{} {}= x-\frac{x^3}{3}-y+1.2 \\ \\ \frac{{\text {d}}y}{{\text {d}}t} &{} {}= 1.25\cdot (0.9-y)+ {\text {UA}}_p(x,y)\end{array}$$			$$\begin{array} {r@{}l@{}} {\text {UA}}_p(x,y) &{} {}= 0.625481 \cdot x,\\ \\ {\text {cf.\;missing\;term}}&{} {}= 0.625\cdot x\end{array}$$	Saddle-Node bifurcation
Bistable FitzHugh–Nagumo	$$\begin{array} {r@{}l@{}}\frac{{\text {d}}x}{{\text {d}}t} &{} {}= x-\frac{x^3}{3}-y+1.0 \\ \\ \frac{{\text {d}}y}{\text {d}t} &{} {}= 1.25\cdot (0.9-y)+ {\text {UA}}_p(x,y)\end{array}$$			$$\begin{array} {r@{}l@{}} {\text {UA}}_p(x,y) &{} {}= 0.624665 \cdot x,\\ \\ \text {cf.\;missing\;term}&{} {}= 0.625\cdot x\end{array}$$	Saddle-Node bifurcation
Monostable Gardner	$$\begin{array} {r@{}l@{}}\frac{{\text {d}}x}{\text {d}t} &{} {}= {\text {UA}}_p(x,y)-x \\ \\ \frac{{\text {d}}y}{{\text {d}}t} &{} {}= \frac{1.5}{1+x^2}-y\end{array}$$			$$\begin{array} {r@{}l@{}} {\text {UA}}_p(x,y) &{} {}= \frac{1.499501}{1+y^2},\\ \\ {\text {cf.\;missing\;term}}&{} {}= \frac{1.5}{1+y^2}\end{array}$$	Pitchfork bifurcation
Bistable Gardner	$$\begin{array} {r@{}l@{}}\frac{{\text {d}}x}{{\text {d}}t} &{} {}= {\text {UA}}_p(x,y)-x \\ \\ \frac{{\text {d}}y}{{\text {d}}t} &{} {}=\frac{3.5}{1+x^2}-y\end{array}$$			$$\begin{array} {r@{}l@{}} \text {UA}_p(x,y) &{} {}= \frac{3.498237}{1+y^2},\\ \\ {\text {cf.\;missing\;term}}&{} {}= \frac{3.5}{1+y^2}\end{array}$$	Pitchfork bifurcation

The nullcline given by the known part of the differential equation is dotted gray and the second nullcline of the true solution is gray dashed.; third column: corresponding time series of the longterm behavior of trained *x* (blue) and *y* (red) variable in comparison to the true solution (black, mainly masked by the predicted time series). The vertical gray line marks the end of the training data; fourth column: corresponding SInDy estimate of ${\text {UA}}_p(x,y)$ based on the approximation of the training data in comparison to the true missing term. The figures are plotted using Julia package Plots (Version v1.16.6, https://github.com/JuliaPlots/Plots.jl) and color-corrected and converted to eps using Adobe Illustrator (Version 25.4.1).
- Pitchfork bifurcation: The Gardner model^31,32^ describes a genetic toggle switch in *Escherichia coli*. In one parametrization, the system is monostable with a stable fixed point but undergoes a Pitchfork bifurcation as the bifurcation parameter decreases, leading to bistable behavior with two stable fixed points and one saddle where each fixed point has its own basin of attraction. (cf. Table 1)
- Hopf bifurcation: The Selkov model^15^ describes oscillatory behavior in the enzyme reactions of the glycolysis process. The systems shows either steady state behavior with a stable fixed point or—undergoing a Hopf bifurcation—ends up in oscillatory behavior with a stable limit cycle. (cf. Table 2)

**Table 2:** Overview of the models used with oscillatory behavior: Selkov model and Rössler model. First column: UDE for the systems in a general *x* and *y* resp. *x*, *y* and *z* notation with $\text {UA}_p(x,y)$ resp. $\text {UA}_p(x,y,z)$ as machine learnable blank; second column: example of a trajectory’s longterm behavior trained with LDA (blue) in state space in comparison to the true solution’s trajectory (black).

	UDE	State space	Time series	SInDy estimate	
Steady state Selkov	$$\begin{array} {r@{}l@{}}\frac{{\text {d}}x}{\text {d}t} &{} {}= - x + {\text {UA}}_p(x,y) + x^2 \cdot y \\ \\ \frac{{\text {d}}y}{{\text {d}}t} &{} {}= 0.15 - 0.1 \cdot y - x^2 \cdot y \end{array}$$			$$\begin{array} {r@{}l@{}} \text {UA}_p(x,y) &{} {}= 0.100014\cdot y, \\ \\ {\text {cf.\;missing\;term}}&{} {}= 0.1 \cdot y\end{array}$$	Hopf bifurcation
Oscillatory Selkov	$$\begin{array} {r@{}l@{}}\frac{{\text {d}}x}{{\text {d}}t} &{} {}= - x + {\text {UA}}_p(x,y) + x^2 \cdot y \\ \\ \frac{{\text {d}}y}{{\text {d}}t} &{} {}= 0.6 - 0.1 \cdot y - x^2 \cdot y \end{array}$$			$$\begin{array} {r@{}l@{}} {\text {UA}}_p(x,y) &{} {}= 0.099735\cdot y, \\ \\ {\text {cf.\;missing\;term}}&{} {}= 0.1 \cdot y\end{array}$$	Hopf bifurcation
Period-one Rössler	$$\begin{array} {r@{}l@{}}\frac{{\text {d}}x}{\text {d}t} &{} {}= -y -z \\ \\ \frac{{\text {d}}y}{{\text {d}}t} &{} {}= x+{\text {UA}}_p(x,y,z) \\ \\ \frac{{\text {d}}z}{{\text {d}}t} &{} {}= 0.1+z\cdot x - 4.0 \cdot z \end{array}$$			$$\begin{array} {r@{}l@{}} {\text {UA}}_p(x,y,z) &{} {}= 0.100181\cdot y, \\ \\ {\text {cf.\;missing\;term}}&{} {}= 0.1 \cdot y\end{array}$$	Period-doubling
Period-two Rössler	$$\begin{array} {r@{}l@{}}\frac{{\text {d}}x}{\text {d}t} &{} {}= -y -z \\ \\ \frac{{\text {d}}y}{{\text {d}}t} &{} {}= x+{\text {UA}}_p(x,y,z) \\ \\ \frac{{\text {d}}z}{{\text {d}}t} &{} {}= 0.1+z\cdot x - 6.0 \cdot z \end{array}$$			$$\begin{array} {r@{}l@{}} \text {UA}_p(x,y,z) &{} {}= 0.100183\cdot y, \\ \\ {\text {cf.\;missing\;term}}&{} {}= 0.1 \cdot y\end{array}$$	Period-doubling

In case of the Selkov model the nullcline given by the known part of the differential equation is dotted gray and the second nullcline of the true solution is gray dashed.; third column: corresponding time series of the longterm behavior of trained *x* (blue) and *y* (red) and in case of Rössler *z* (cyan) variable in comparison to the true solution (black, mainly masked by the predicted time series). The vertical gray line marks the end of the training data; fourth column: corresponding SInDy estimate of ${\text {UA}}_p(x,y)$ resp. ${\text {UA}}_p(x,y,z)$ based on the approximation of the training data in comparison to the true missing term. The figures are plotted using Julia package Plots (Version v1.16.6, https://github.com/JuliaPlots/Plots.jl) and color-corrected and converted to eps using Adobe Illustrator (Version 25.4.1).
- Period-doubling: The Rössler model^33,34^ can describe chemical reactions, but is often chosen as example for a simple period-doubling cascade. Here we focus on the transition from a period-one limit cycle to a period-two limit cycle with changing parametrization. (cf. Table 2)

We wanted to evaluate the performance of a UDE trained with the LDA loss function against a UDE trained with the MSE loss function for each model with two different parametrizations leading to the two different dynamical behaviors of the systems named above. Furthermore, we considered three different levels of normal distributed noise added to the training data to test the robustness of our results under more realistic conditions.

The universal function approximator ${\text {UA}}_p(x,y)$ (Equation 1) in all examples was a neural network with a single hidden layer and 16 neurons with *tanh*-activation function to accommodate a wide variety of possible non-linear functions with a limited number of ANN parameters. The initial weights of the Neural Network are chosen randomly using the Glorot initializer^35^ with a Normal Distribution as its basis. We used the ADAM optimizer^36^ and added weight decay to train the UDE (see Supplementary Table S1 for additional details).

Tables 1 and 2 show representative results of the longterm prediction of the UDE model trained with LDA, where four times the length of the training data is used.

To compare estimated trajectories of the UDE trained with LDA or MSE loss objectively, we calculated the trajectory difference (TD) as the sum over the length of difference vectors over the sample points of the UDE and samples from the full differential equation:

4 $$\begin{aligned} TD= & {} \frac{1}{n}\cdot \sum _{i=1}^{n} ||\mathbf{x }_{i}-\mathbf{p }_{i}||, \end{aligned}$$

where the true trajectory is described by the position vectors of the *n* trajectory points $x_i$ and the approximated position vectors of the *n* trajectory points $p_i$.

Because the initial weights $w_{\text {init}}$ of the machine learning model can considerably determine the success of UDE training (cp. Fig. 2), we independently drew 100 different sets of initial weights $w_{\text {init}}$ per model, model parametrization and loss function and calculated TD for each independent experiment.

**Figure 3:**
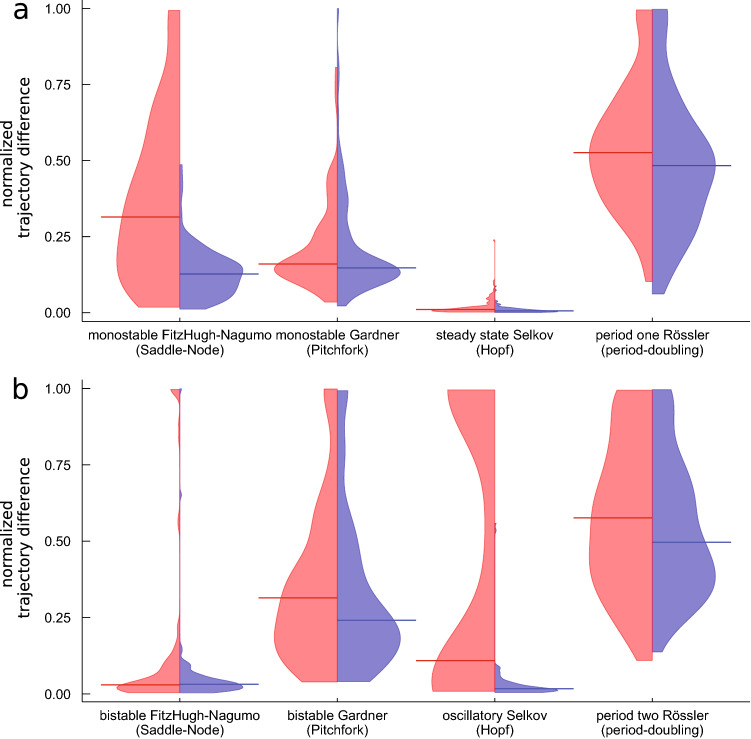
Distribution of the trajectory difference (TD) for the longterm prediction of the bifurcating systems over 100 neural network initializations trained using MSE loss function (red) or LDA loss function (blue). The horizontal bar indicates the median of the respective distribution. The training data contain additive normal distributed noise of noise level 0.0001. The trajectory difference is normalized by TD = 1.0 for FitzHugh–Nagumo, by TD = 0.1 for the Gardner, by TD = 1.0 for the Selkov and by TD = 2.0 for the Rössler model. All normalized values larger than one are clipped to one. The starting positions of the trajectories are chosen as for the examples in Tables 1 and 2. (**a**) Monostable, steady state and period-one parametrization (**b)** bistable, oscillatory and period-two parametrization. This figure is plotted using Julia package Plots (Version v1.16.6, https://github.com/JuliaPlots/Plots.jl) and color-corrected and converted to eps using Adobe Illustrator (Version 25.4.1).

In Fig. 3 we show the distribution of a normalized version of TDs we get over all these 100 trained UDEs to compare the quality of the UDE solutions across different systems, parametrizations and loss function used in one figure. The general form of these distributions remains stable across all noise levels. All results for different noise levels can be found in Supplementary Figs. S1 and S2.

#### Systems with a changing number of fixed points

The Pitchfork and Saddle-Node bifurcations lead to a changing number of fixed points in the Gardner and FitzHugh–Nagumo systems, respectively. A good approximation of the system’s longterm behavior, indicated by small TD between the approximated and the true solution’s trajectory, is in general possible with both loss functions (see Fig. 3).

However, for the FitzHugh–Nagumo model, the results using the MSE as the loss function show a higher median compared to the LDA loss and a larger distribution width in case of the monostable parametrization (cf. Fig. 3a). The reason why is that the predicted trajectories vary more around the true solution’s trajectory which leads to a number of trajectories ending in alternative stable states along the given nullcline (see Supplementary Fig. S3c,d). This effect is also visible in the outliers of the TD distribution for the bistable case for the MSE loss function, which make up around 12% of all trajectories end up in alternative stable states along the nullcline given by the known part of the differential equation in the region of the second fixed point (cf. Fig. 3b and Supplementary Fig. S3b). This second stable fixed point is also the only stable fixed point in case of the monostable parametrization (cf. Tabel 1). The LDA loss function does not lead to this behavior, because the angular difference between trajectories favors the correct curvature of the trajectory compared to absolutely correct values and guides the trajectory during training towards the correct fixed point. This reflects more precisely what we hope to achieve with the learned UDE, and leads to a compact TD distribution for the longterm prediction. Only around 2% of the trajectories in the bistable case end in the alternative stable state (cf. Fig. 3b and Supplementary Fig. S3a).

The same but somewhat weakened effect is observed for the bistable Gardner model (cf. Fig. 3b). Here, the results using the MSE loss function show 6–7% of the predicted longterm trajectories terminating in the alternative stable state on the given nullcline close to the fixed point the true solution’s trajectory ends up in (cf. Table 1 and Supplementary Fig. S4b). The results using the LDA loss function only show up to 5% of the trajectories ending in alternative stable states (see Supplementary Fig. S4a).

The main difference in the bistable parametrization of the FitzHugh–Nagumo compared to the bistable Gardner model is, that the alternative states in the Gardner model are not linked to either the second stable fixed point or the stable fixed point in the monostable parametrization. Rather, they only occur spuriously in the functions permitted by the UDE in its current parametrization. The underlying dynamical structure of the Gardner system separates the state space into regions that specify to which fixed point all trajectories starting in this region converge. This dynamical behavior cannot be broken during the training. The alternative stable states can likely be linked to a new parametrization of the bistable Gardner system that co-evolves during training of the UDE.

In case of the monostable parametrization of the Gardner system, both loss functions lead to approximately equally good approximations of the longterm behavior of the system (cf. Fig. 3a and Supplementary Fig. S4c,d).

#### Systems with oscillatory behavior

The Hopf and period-doubling bifurcation lead to changing dynamical behavior in the Selkov and Rössler systems, from non-oscillatory to oscillatory behavior or from period one to period two oscillatory behavior respectively.

For steady state behavior of the Selkov system, both loss functions lead to good approximations of the longterm behavior of the system without strong dependence on the noise level, with a median TD of around 0.006 for the LDA loss function and around 0.012 for the MSE (cf. Fig. 3a and Supplementary Fig. S5c,d). By contrast, for the case of the oscillatory parametrization of the Selkov system, the results using the MSE loss function have a bimodal TD distribution, whereas the trajectory-based LDA loss function still leads to a narrow TD distribution and consistently good approximations (cf. Fig. 3b).

This is because many initial conditions in the MSE case lead to an alternative steady state solution during optimization which is a local minimum on the loss function that cannot be left (cf. Fig. 2b and Supplementary video material S1 panel b). The bimodal nature of the TD distribution reflects this: if the initial condition is favorable, the learning process finds the correct solution, if it is not, it fails completely (see supplemental Fig. S5b). The calculated median of the TD distribution shifts depending on the number of successful approximations and complete failures (see Supplementary Fig. S2b).

The LDA loss function on the other hand benefits from the combination of angular loss and the vector length loss, which allow for detours in state space (cf. Supplementary video material S1 panel a) bringing the approximated solution during training in the region of the training data. The solutions reliably reproduce the correct oscillations of the original system in nearly all cases (see Supplementary Fig. S5b).

For the case of the Rössler system, the approximations of the training data for both parametrizations show median values of TD around 0.04 for both parametrizations for the MSE and around 0.03 for both parametrizations for the trajectory-based loss function. The distribution is compact around the median with a few outliers (see Supplementary Fig. S6). By contrast, the longterm prediction of both used loss functions show median values of TD around 1.04 (period-one parametrization) resp. 1.22 (period-two parametrization) for MSE and around 0.95 (period-one parametrization) resp. 1.10 (period-two parametrization) for the LDA loss function (cf. Fig. 3a,b). Only a few runs yield good approximations. The reason why is that the training data do not include the typical excursions of the Rössler system in z-direction in state space which are included in the longterm prediction (cf. Table 2). These excursions are not approximated well by either of the trained networks. Longer training data could improve these results. Nevertheless, the accurate approximation of the short term training data enables a proper reconstruction of the algebraic form of the missing term as in Table 2.

### The loss functions around the bifurcation

**Figure 4:**
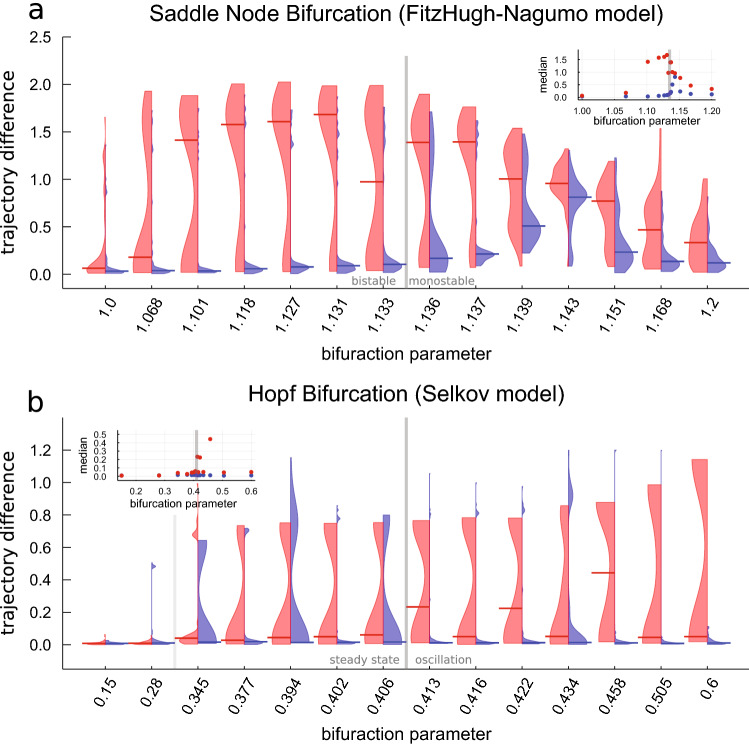
Distribution of the trajectory difference (TD) for the longterm prediction of the bifurcating systems over 50 neural network initializations trained using the MSE loss function (red) or LDA loss function (blue) for FitzHugh–Nagumo (**a**) and Selkov model (**b**) around the bifurcation (gray bar). The light gray bar in case of the Selkov model indicates the onset of strongly damped oscillations. The horizontal bar indicates the median of the respective distribution. The median of the distribution is plotted in the inset. The training data contain additive normal distributed noise with the noise level set to 0.0001. The trajectory difference larger than 1.2 are clipped to 1.2 in case of the Selkov model (**b**). The bifurcation parameter in case of FitzHugh–Nagumo is the 4th term in the first equation in Table 1 and in case of Selkov the first term in the second equation in Table 2. The trajectories start at $(-2.0,-0.25)$ in case of FitzHugh–Nagumo and at (1.0, 1.0) in case of Selkov. This figure is plotted using Julia package Plots (Version v1.16.6, https://github.com/JuliaPlots/Plots.jl) and color-corrected and converted to eps using Adobe Illustrator (Version 25.4.1).

In the previous section we observed very different distributions for the trajectory difference depending on the chosen loss function and the parametrizations of the systems. To investigate if the distributions show a gradual change from one type to the other across the bifurcation, we performed 50 training runs for the FitzHugh–Nagumo and the Selkov model each with 14 different parametrization chosen from an interval nesting of the bifurcation parameter around the bifurcation. The bifurcation parameter for the case of FitzHugh–Nagumo is the 4th term in the first equation in Table 1 and in case of Selkov the first term in the second equation in Table 2. The FitzHugh–Nagumo and the Selkov model were chosen because they show the most different distributions of the trajectory difference TD for the two different dynamical behaviors (cf. Fig. 3). The results are presented in Fig. 4.

Firstly, we see a change of the shape of the MSE loss distribution as soon as the curvature of the trajectory increases (FitzHugh–Nagumo model Fig. 4a) or as soon as strongly damped oscillations start (Selkov model Fig. 4b between the light gray and the gray bar). The median of the LDA TD distribution is only sensitive to this in a narrow band of parameters shortly after the bifurcation in the FitzHugh–Nagumo model (cf. inset in Fig. 4a). Thus, the distributions reflect the bifurcation, too. As the curvature of the trajectory changes drastically across the bifurcation for both systems, the difference in the quality of the approximation using either loss function is markedly different. The reason for this behavior is that MSE only optimizes the difference in each variable in state space independently. This approach misses differences in the curvature of the training data trajectory and the current approximated solution. Thus, the MSE is often not suited to approximate a proper solution for a randomly chosen first guess of the approximated solution that belongs to a different dynamical regime than the training data and must be transferred to another dynamical regime during training by temporarily changing the curvature of the approximated solution drastically.

In contrast, the LDA loss function indirectly contains information about the curvature difference in its angle part of the loss function at a specific position in state space (length difference part of the loss function). Therefore, the LDA loss can capture changes in curvature earlier and permits the randomly chosen first guess UDE in the wrong dynamical regime to change to the dynamical regime of the training data more reliably. On the other hand considering small changes in the direction of a trajectory can immediately cause bad performance of LDA for specific noisy trajectory passing very close to a saddle. These small changes in the direction of the trajectory can lead to a switch of the dynamical behavior and a broader TD distribution (cf. Fig. 4a parameter 1.139 and 1.143).

In sum, there is an abrupt change from one type of distribution to another around the bifurcation reflecting the ability of the loss functions to reliable capture changes in curvature or not.

### The loss functions and different starting points of trajectories

**Figure 5:**
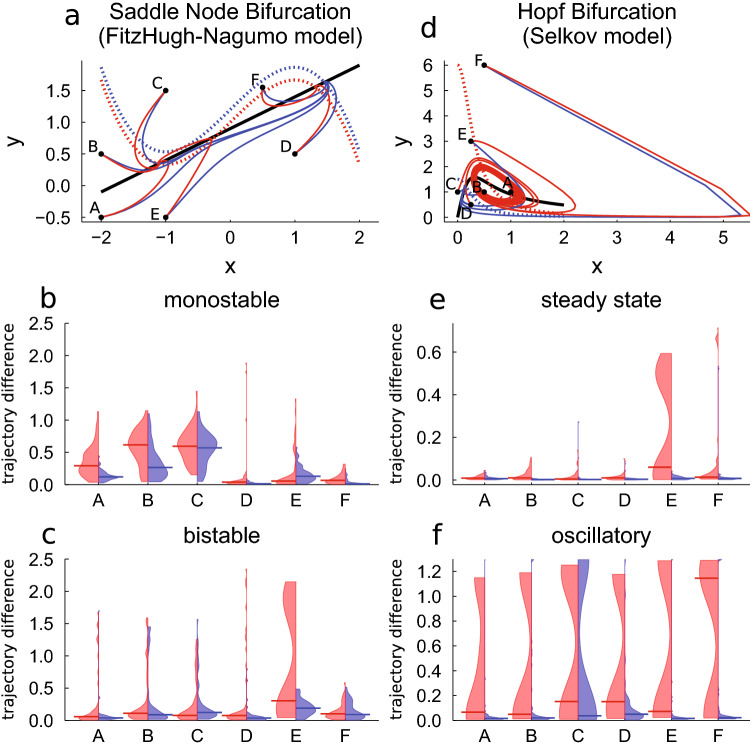
Distribution of the trajectory difference (TD) for the longterm prediction of the FitzHugh–Nagumo model (**b**, **c**) and the Selkov model (**e**, **f**) over 50 neural network initializations trained using the MSE loss function (red) or LDA loss function (blue) for starting position A to F of the trajectories (**a**, **d**) using two different parametrizations. The horizontal bar indicates the median of the respective distribution. The training data contain additive normal distributed noise with the noise level set to 0.0001. The red trajectories correspond to the bistable case (**a**) resp. oscillatory case (**d**), the blue one to the monostable case (**a**) resp. steady state (**d**). The black curve is the nullcline given by the known part of the differential equation, the dashed red (blue) curve is the second nullcline corresponding to the bistable resp. oscillatory state (to the monostable resp. steady state) in (**a**) resp. (**b**). This figure is plotted using Julia package Plots (Version v1.16.6, https://github.com/JuliaPlots/Plots.jl) and color-corrected and converted to eps using Adobe Illustrator (Version 25.4.1).

To investigate the impact of the starting position of the trajectories in state space on the performance of a UDE trained with the LDA loss function against a UDE trained with the MSE loss function, we again trained 50 UDEs for the FitzHugh–Nagumo and the Selkov model in the two parametrizations used in Tables 1 and 2 respectively. We chose starting points with very different dynamical behavior of the trajectories, namely slow and fast dynamics in state space as well as weak and strong curvatures of the trajectories. The results are presented in Fig. 5.

For both models we observe a strong dependence of the success of the training on the starting position in state space when using MSE. Less successful approximations are often linked with strong curvatures or oscillatory behavior. In case of the LDA loss function for the monostable FitzHugh–Nagumo model we observe less successful training for starting position B, C and E (cf. Fig. 5b). B and C share the common feature that they pass very slowly close to the saddle and the training data trajectory ends in this region, thus small changes of the trajectory due to noise can lead to a broadening of the distribution of the longterm prediction. Starting position E corresponds to a trajectory with a fast dynamics in case of the monostable parametrization and an extremely curved trajectory in case of the bistable parametrization. While we observe a wider distribution of trajectory differences in the monostable case, the LDA loss still captures the correct dynamical regime in case of the bistable parametrization and does not converge to a different fixed point as is possible for the MSE (cf. Fig. 5c).

When learning the Selkov model, the MSE shows a bimodal distribution for the oscillatory parametrization as expected, but this is also the case for starting point E in the steady state parametrization (cf. Fig. 5e). E is very close to second nullcline in state space which is unknown for the UDE. One possible explanation is that during training two different solution classes (steady state and oscillatory) are realized. We even observe a bimodal distribution in case of the LDA for the steady state and the oscillatory parametrization if we apply the standard values for the factors $k_1$ and $k_2$ used in the experiments above. If we adjust $k_1$ to 0.1 and $k_2$ to 0.9, the bimodal distribution vanishes and successful training is possible (cf. E in Fig. 5e,f). The same effect occurs for D in case of the oscillatory parametrization (cf. Fig. 5f), whereas a change of $k_1$ and $k_2$ does not impact the results for C (cf. Fig. 5f).

To sum up, the starting position of the trajectory and its resulting properties can impact the success of the training. This is especially the case for the MSE, where there are no further hyperparameters to tune the solution.

### SInDy’s answer: algebraic form of the missing term

Finally, to get an idea of the algebraic form of the missing term, an approach for sparse identification of nonlinear dynamical systems (SInDy)^12^ by Brunton et al. was applied to examples trained with the LDA loss function. This approach was chosen to show by way of example that a successful trained UDE system can be translated into an algebraic form. The examples are chosen from the statistical analysis with the lowest noise level such that the trajectory difference between the approximated solution of the training data and the training data itself is smallest. We investigated one example for each parametrization and each model. To improve the performance of SInDy, outliers in the beginning and the end of the approximated guess of the dynamics of the missing term are removed before SInDy is applied. The optimizer used by SInDy is STRRidge^12^. The basis functions from which SInDy can select its guess are polynomials up to order of 5, except for the case of the Gardner model where polynomials up to order of 3 are combined with terms of the form $\frac{1}{1+z^n}$ with *z* being either *x* or *y* and $n=1,2,3$. This exception was chosen to identify the term more clearly and not hidden it in a polynomial expansion of the function.

The results are shown in Tables 1 and 2. All in all, the algebraic form of the missing terms as well as the system parameters are approximated well.

The approach is not limited to the examples shown. It is possible to learn other terms or several terms at the same time. A crucial precondition for a successful reconstruction of the missing term with SInDy is an accurate approximation of the dynamics of the missing term with the UDE approach, which is directly linked with the successful reconstruction of the dynamical behavior of the training data. Thus, the success of the SInDy approach is directly linked with the accuracy of the results of the UDE approach. Furthermore, an accurate reconstruction of the missing term with SInDy based only on the approximation of the training data might give better results for the longterm prediction of the dynamics than the UDE, because the sparsity criterion of SInDy suppresses numerical fluctuations present in the UDE that might interfere with the longterm prediction.

## Discussion and conclusion

In summary, the LDA loss function leads to more stable and accurate approximation of the longterm dynamical behavior in systems with a changing number of fixed points or a change in oscillatory behavior due to a bifurcation.

Often, trained networks using MSE as loss function lead to a solution which is only slightly worse than the networks using the LDA loss function, but there is a risk of complete failure of the approximation depending on the network initialization. Local minima in the MSE loss function due to different dynamical regimes of the UDE can represent completely different dynamical solutions, which gradient-based learning cannot recover from by adapting the gradient-descent procedure with, for example, momentum^37^ or adaptive learning rates^36^.

We used the LDA loss, which optimizes two components, the length and angle of state space vectors, with different local minima. A weighted combination of both components allowed us to estimate the dynamics of bifurcating systems more accurately, and avoided the catastrophic scenario of local minima leading to different dynamic regimes. While some randomly chosen initial parameters for the neural network can still lead to a bad fit of the UDE, we found that in examples where the MSE loss often finds local minima in the wrong dynamic regime, the basin of attraction of the optimization process was indeed much larger when we used the LDA loss instead. In some cases the choice of the factors weighting the two components can improve the results of the training as shown in case of the study of different starting points. We demonstrated that in applications, the loss function has to be chosen with great care based on the dynamics of the measured trajectories. This is because there is no gradual change from the possibility of successful training to failure across the bifurcation.

A more general open questions of UDE is the impact of the training data length and its temporal resolution on the successful approximation of the system. A preliminary survey of this question for examples of the LDA loss does not indicate a clear effect for the temporal resolution (cf. supplementary Fig. S7) but as expected the trajectory differences between the approximated and the true solution trajectory decreases with increasing training data length (cf. supplementary Fig. S8). A more comprehensive study should be the content of further research, because in any real data set of measured time series the length and temporal resolution is fixed.

In real-world applications, physics-informed neural network approaches have successfully been applied to, among others, fluid dynamical problems^38,39^. SInDy has also shown good results in complex data-driven settings^40–42^. Universal Differential Equations^11^ elegantly bring together two important aspects of these approaches: the advantages of knowledge about the underlying physics in the form of a differential equation, and the statistical estimation of dynamics via machine learning. SInDy^12^ can be used to open the black box of machine learning and reconstruct algebraic terms to complete a differential equation. This approach to knowledge discovery is powerful. It implies that some interactions in the system of interest are not yet discovered, which is particularly true for—for example—ecosystems that cannot completely be examined in the lab or for systems for which the underlying governing equation is unknown. In such systems tipping points and potentially unknown bifurcations are of particular interest.

We have demonstrated, that UDEs can be used to estimate and reconstruct missing terms even in bifurcating systems. However, all examples shown here are simple compared to real-world applications. But even at this basic level we only have a rough idea of the interaction of the learning process and the bifurcation properties of the system. The interaction of both the dynamics of gradient-descent optimizing a loss function and the dynamics of the system of interest itself is highly fascinating and not yet understood. At this stage more fundamental research is necessary.

Particular care has to be taken in how the loss function is constructed for the optimization process, because the optimization process itself can lead to bifurcations. Nonetheless, our results suggest that dynamics in real-world, not yet fully described systems can be discovered using the LDA loss function.

## Supplementary Information

Supplementary Movie S1.

Supplementary Information.

## Data Availability

All experiments have been implemented in the Julia programming language^13^ based on packages developed particularly to fit UDEs with neural networks (https://github.com/SciML/DiffEqFlux.jl) and SInDy (https://github.com/SciML/DataDrivenDiffEq.jl). The code to produce all data presented in the paper is available publically at https://github.com/pnieters/GeneralizedDynamicsFromData.

## Code availability

*Accession codes* The code to produce all data presented in the paper is available publically at https://github.com/pnieters/GeneralizedDynamicsFromData.
