# Supplementary figures and images for: A trajectory-based loss function to learn missing terms in bifurcating dynamical systems
Source: Sci Rep. 2021 Oct 14;11:20394. doi: 10.1038/s41598-021-99609-x (PMC8516982; doi:10.1038/s41598-021-99609-x)

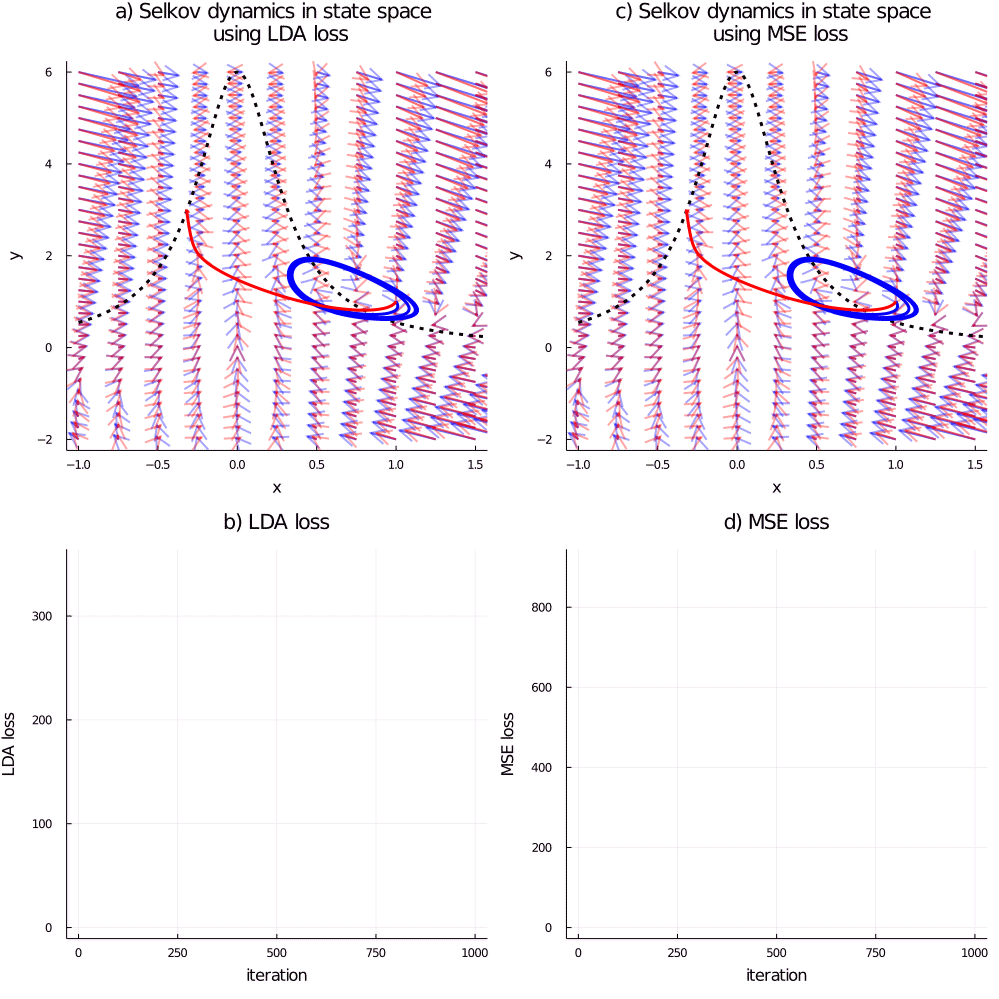

Supplement: Supplementary file 1 — Supplementary Movie S1. [file 41598_2021_99609_MOESM1_ESM.gif]
