# Supplementary material for: A trajectory-based loss function to learn missing terms in bifurcating dynamical systems
Source: Sci Rep. 2021 Oct 14;11:20394. doi: 10.1038/s41598-021-99609-x (PMC8516982; doi:10.1038/s41598-021-99609-x)
Supplement: Supplementary file 2 — Supplementary Information. [file 41598_2021_99609_MOESM2_ESM.pdf]

Supplementary Material to: A trajectory-based loss function to learn missing terms in bifurcating dynamical systems

Rahel Vortmeyer-Kley[1,*,+], **Pascal Nieters**[1,+], **and Gordon Pipa**[1]

[1]Institute of Cognitive Science, Osnabrück University, Wachsbleiche 27, 49090 Osnabrück, Germany
[*]rahel.vortmeyer-kley@uni-osnabrueck.de
[+]these authors contributed equally to this work

ABSTRACT

This supplementary material contains additional figures to the paper: "A trajectory-based loss function to learn missing terms in bifurcating dynamical systems"

Table S1. Configuration of the ADAM optimizer in all experiments used in the code:

Model (loss function)	Learning Rate (LR)	LR Decay Rate	Decay Steps	LR Minimum	Weight Decay
FitzHugh-Nagumo (LDA)	1.00E-01	0.5	100	1.00E-04	1.00E-04
FitzHugh-Nagumo (MSE)	1.00E-01	0.5	100	1.00E-04	1.00E-04
Gardner (LDA)	1.00E-02	0	0	0	0
Gardner (MSE)	1.00E-02	0	0	0	0
Selkov (LDA)	1.00E-02	0.5	800	1.00E-04	1.00E-04
Selkov (MSE)	1.00E-02	0.5	800	1.00E-04	1.00E-04
Rössler (LDA)	1.00E-01	0.5	100	1.00E-04	1.00E-04
Rössler (MSE)	1.00E-01	0.5	100	1.00E-04	1.00E-04

Caption to Movie S1 (separate .gif-file).
Evolution of the current estimated trajectory (red) for the oscillatory Selkov system and its corresponding velocity field (red quivers) in comparison to the training data (blue) and its corresponding velocity field (blue quiver):
panel a): training with LDA loss;
panel c): training with MSE loss.
The black dotted line is the nullcline given by the known part of the differential equation. The lower two panels show the evolution of the respective loss during training (Panel b): LDA loss and panel d): MSE loss).
The underlying system does not contain additive noise and is trained with a simplified network setup of two input and one output-neuron without hidden layers to keep it computational cheap.

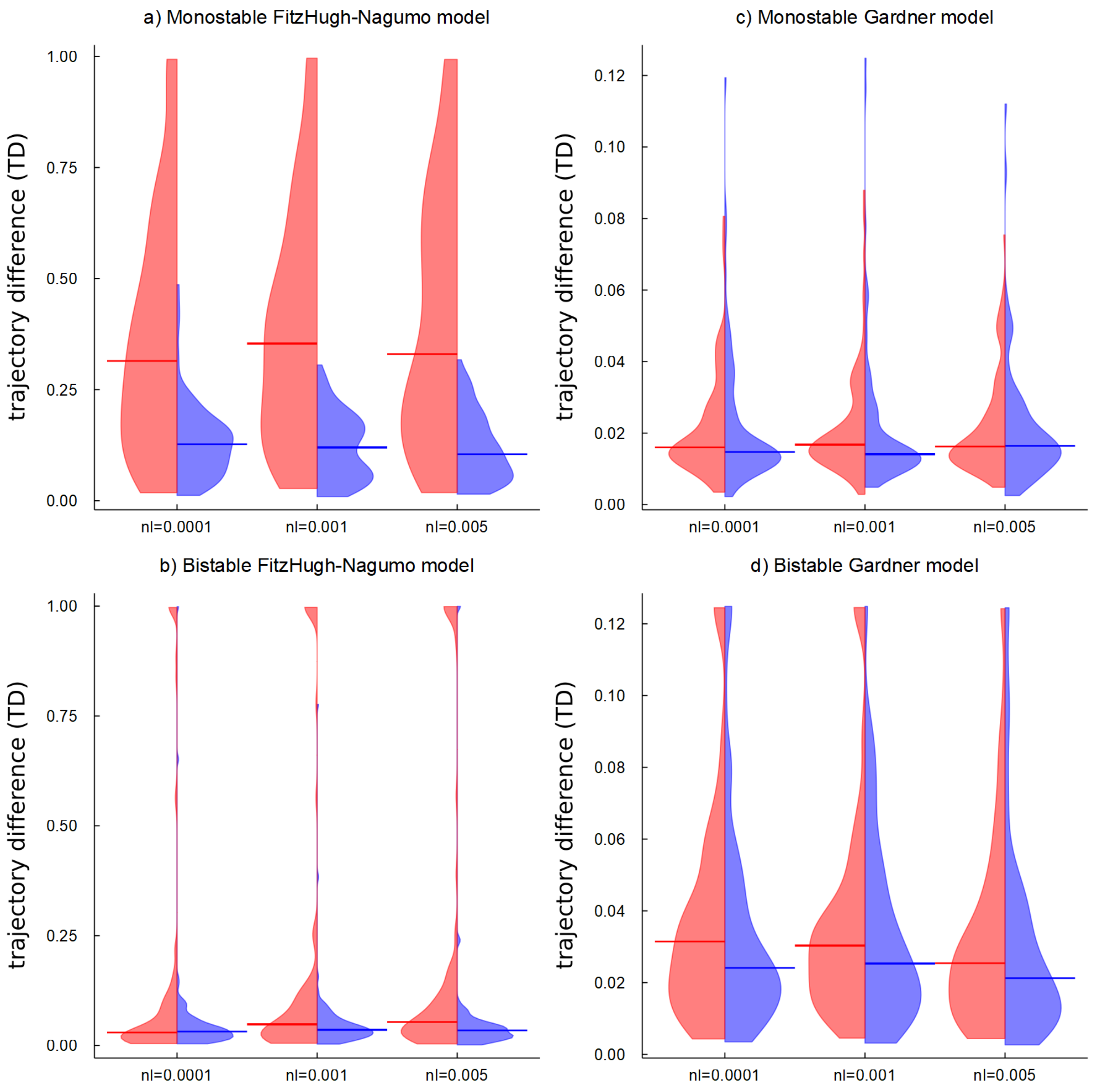

Figure S1. Distribution of the trajectory difference (TD) for the longterm prediction of systems with changing number of fixed points trained using MSE loss function (red) or LDA loss function (blue). The horizontal bar indicate the median of the respective distribution. The training data contain additive normal distributed noise of noise level nl.
a) FitzHugh-Nagumo model in monostable parametrization, b) FitzHugh-Nagumo model in bistable parametrization; all TD values larger than 1.0 are clipped to 1.0.
c) Gardner model in monostable parametrization, d) Gardner model in bistable parametrization; all TD values larger than 0.125 are clipped to 0.125.
This figure is plotted using Julia package Plots (Version v1.16.6, `https://github.com/JuliaPlots/Plots.jl`).

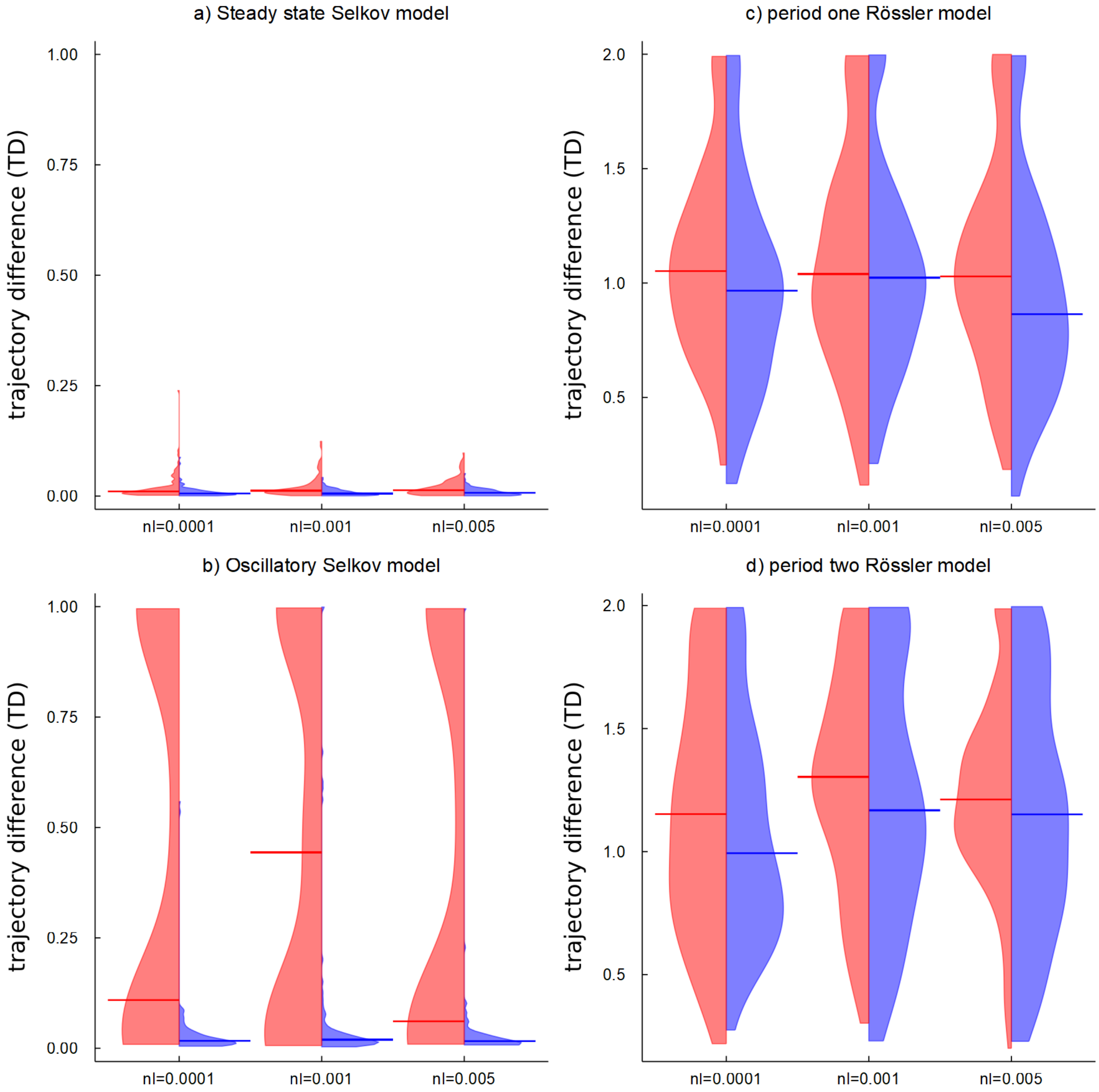

Figure S2. Distribution of the trajectory difference (TD) for the longterm prediction of systems with oscillatory behavior trained using MSE loss function (red) or LDA loss function (blue). The horizontal bar indicate the median of the respective distribution. The training data contain additive normal distributed noise of noise level nl.
a) Selkov model in steady state parametrization, b) Selkov model in oscillatory parametrization; all TD values larger than 1.0 are clipped to 1.0.
c) Rössler model in period one parametrization, d) Rössler model in period two parametrization; all TD values larger than 2.0 are clipped to 2.0.
This figure is plotted using Julia package Plots (Version v1.16.6, https://github.com/JuliaPlots/Plots.jl).

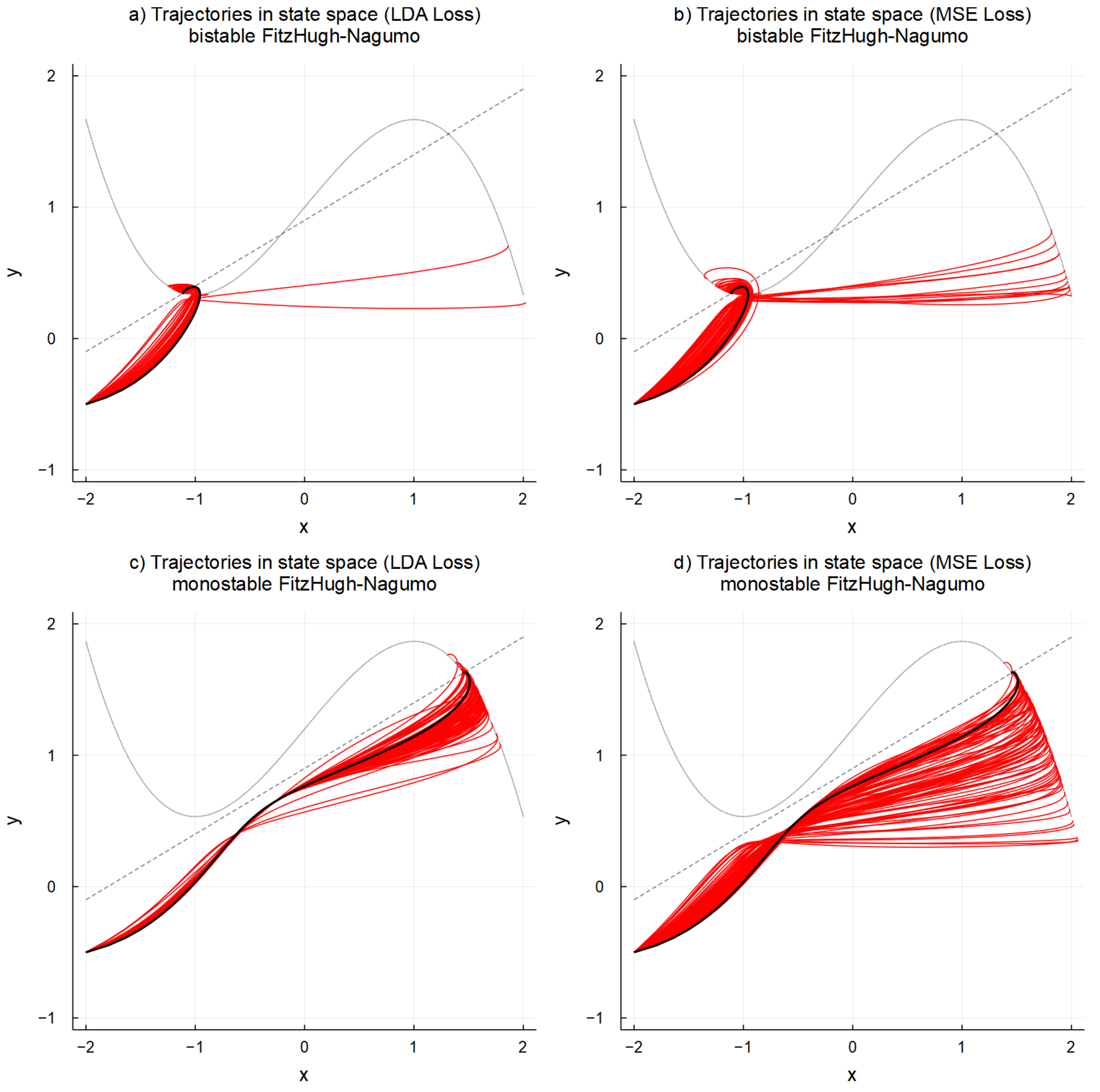

Figure S3. State space plot of the trajectories of the statistical experiment performed on the FitzHugh-Nagumo model with two different parametrizations and the two different loss functions for the noise level nl=0.0001: The plot contains 100 trajectories of the longterm prediction in state space (red) each, that were trained using the MSE or the LDA loss in comparison to the true solution (black) for the bistable parametrization (a) LDA loss and b) MSE loss) and for the monostable parametrization (c) LDA loss and d) MSE loss). The gray dotted line is the nullcline given by the known part of the differential equation of the system, the gray dashed line is the second nullcline of the true system.
This figure is plotted using Julia package Plots (Version v1.16.6, `https://github.com/JuliaPlots/Plots.jl`).

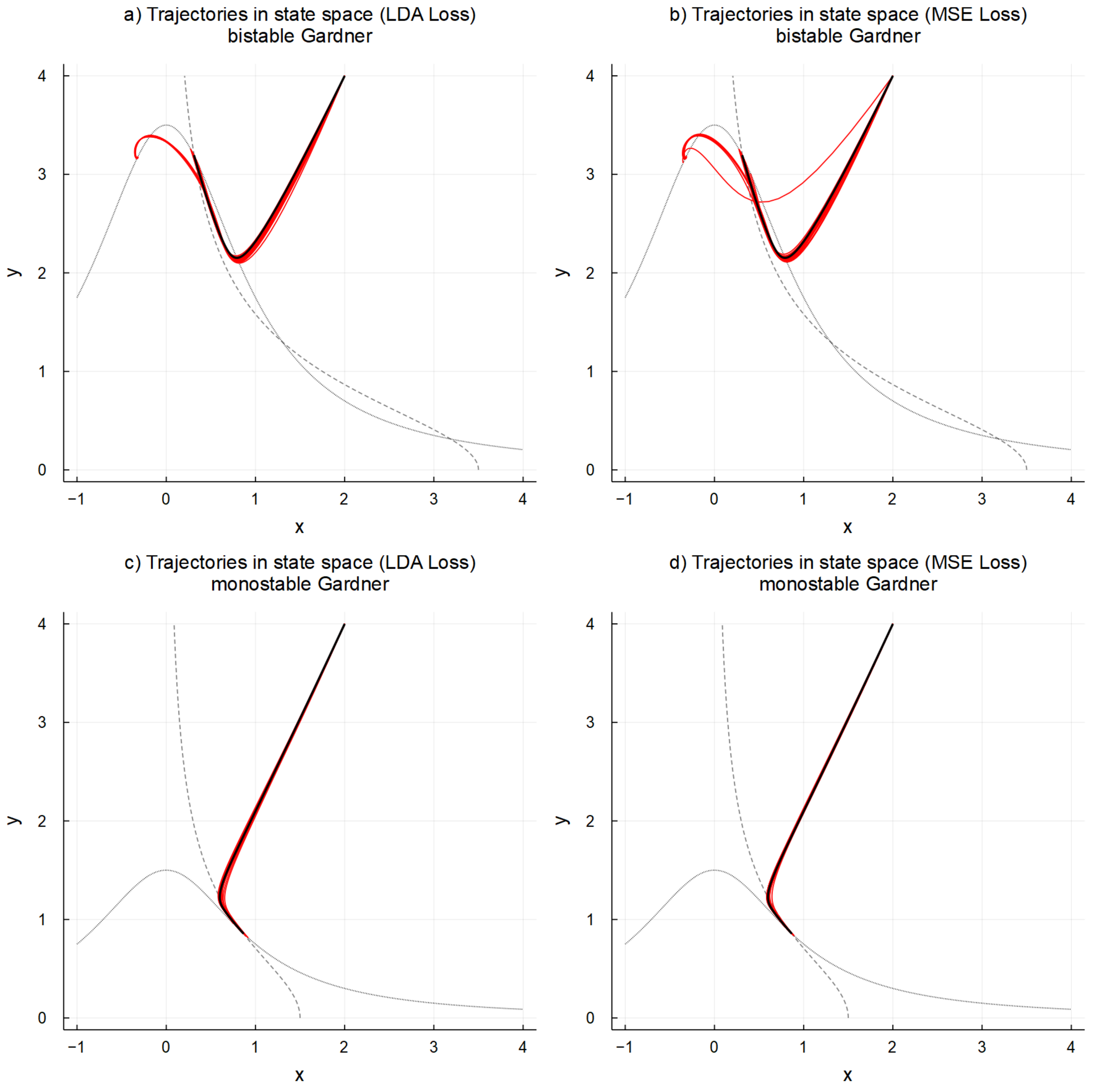

Figure S4. State space plot of the trajectories of the statistical experiment performed on the Gardner model with two different parametrizations and the two different loss functions for the noise level nl=0.0001: The plot contains 100 trajectories of the longterm prediction in state space (red) each that were trained using the MSE or the LDA loss in comparison to the true solution (black) for the bistable parametrization (a) LDA loss and b) MSE loss) and for the monostable parametrization (c) LDA loss and d) MSE loss). The gray dotted line is the nullcline given by the known part of the differential equation of the system, the gray dashed line is the second nullcline of the true system.
This figure is plotted using Julia package Plots (Version v1.16.6, `https://github.com/JuliaPlots/Plots.jl`).

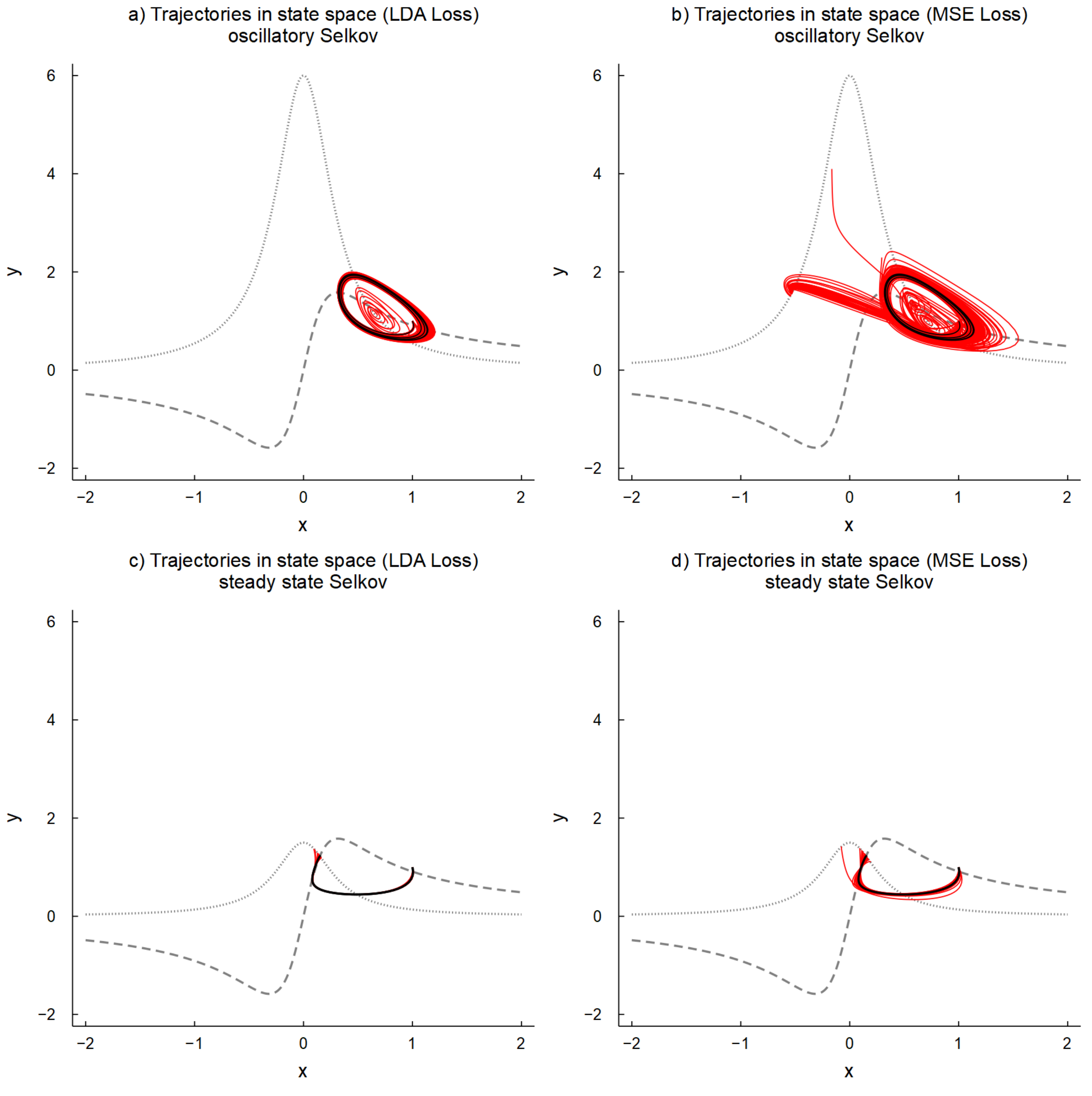

Figure S5. State space plot of the trajectories of the statistical experiment performed on the Selkov model with two different parametrizations and the two different loss functions for the noise level nl=0.0001: The plot contains 100 trajectories of the longterm prediction in state space (red) each that were trained using the MSE or the LDA loss in comparison to the true solution (black) for the oscillatory parametrization (a) LDA loss and b) MSE loss) and for the steady state parametrization (c) LDA loss and d) MSE loss). The gray dotted line indicate the nullcline given by the known part of the differential equation of the system, the gray dashed line is the second nullcline of the true system.
This figure is plotted using Julia package Plots (Version v1.16.6, https://github.com/JuliaPlots/Plots.jl).

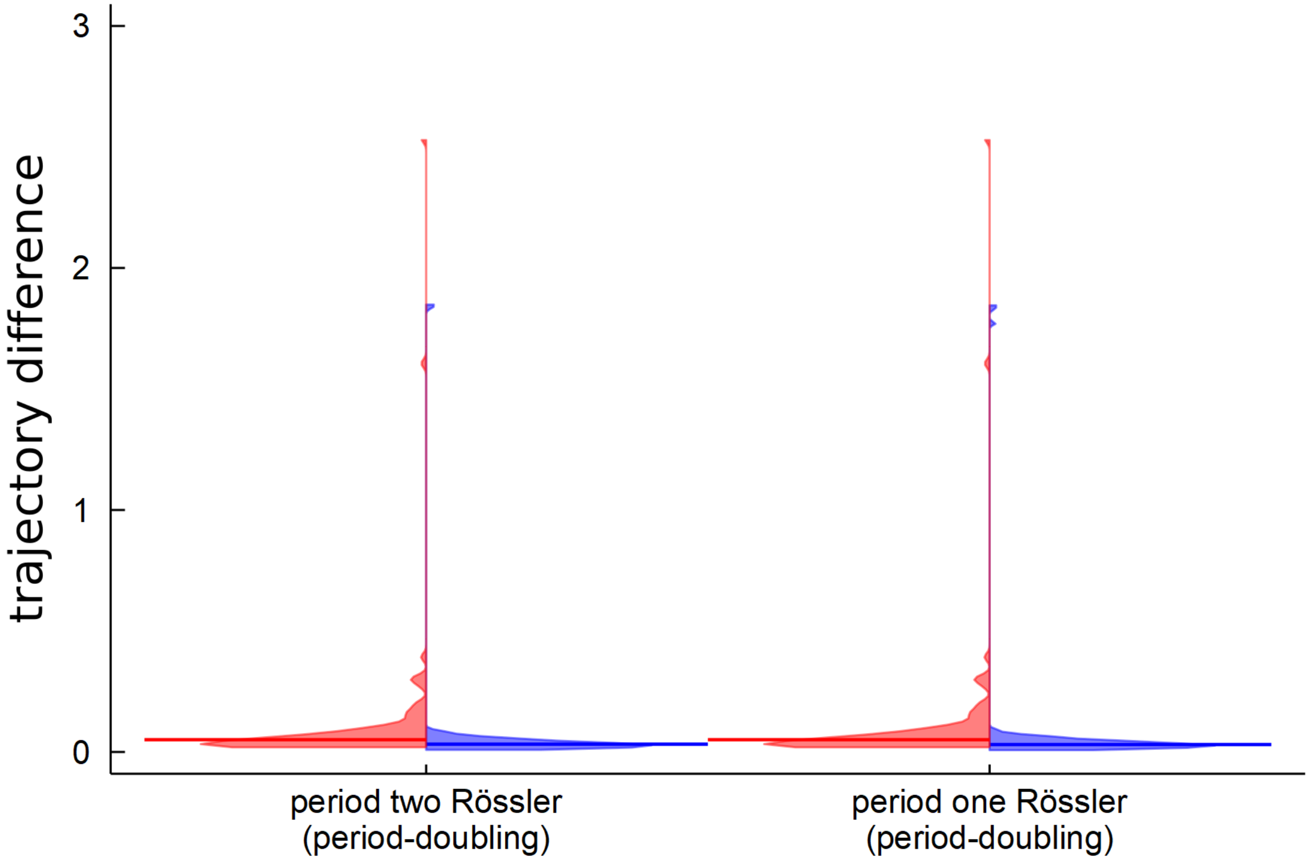

Figure S6. Distribution of the trajectory difference (TD) for the prediction of the training data of the Rössler system trained using MSE loss function (red) or LDA loss (blue). The horizontal bar indicate the median of the respective distribution. The training data contain additive normal distributed noise of noiselevel nl=0.0001.
This figure is plotted using Julia package Plots (Version v1.16.6, `https://github.com/JuliaPlots/Plots.jl`).

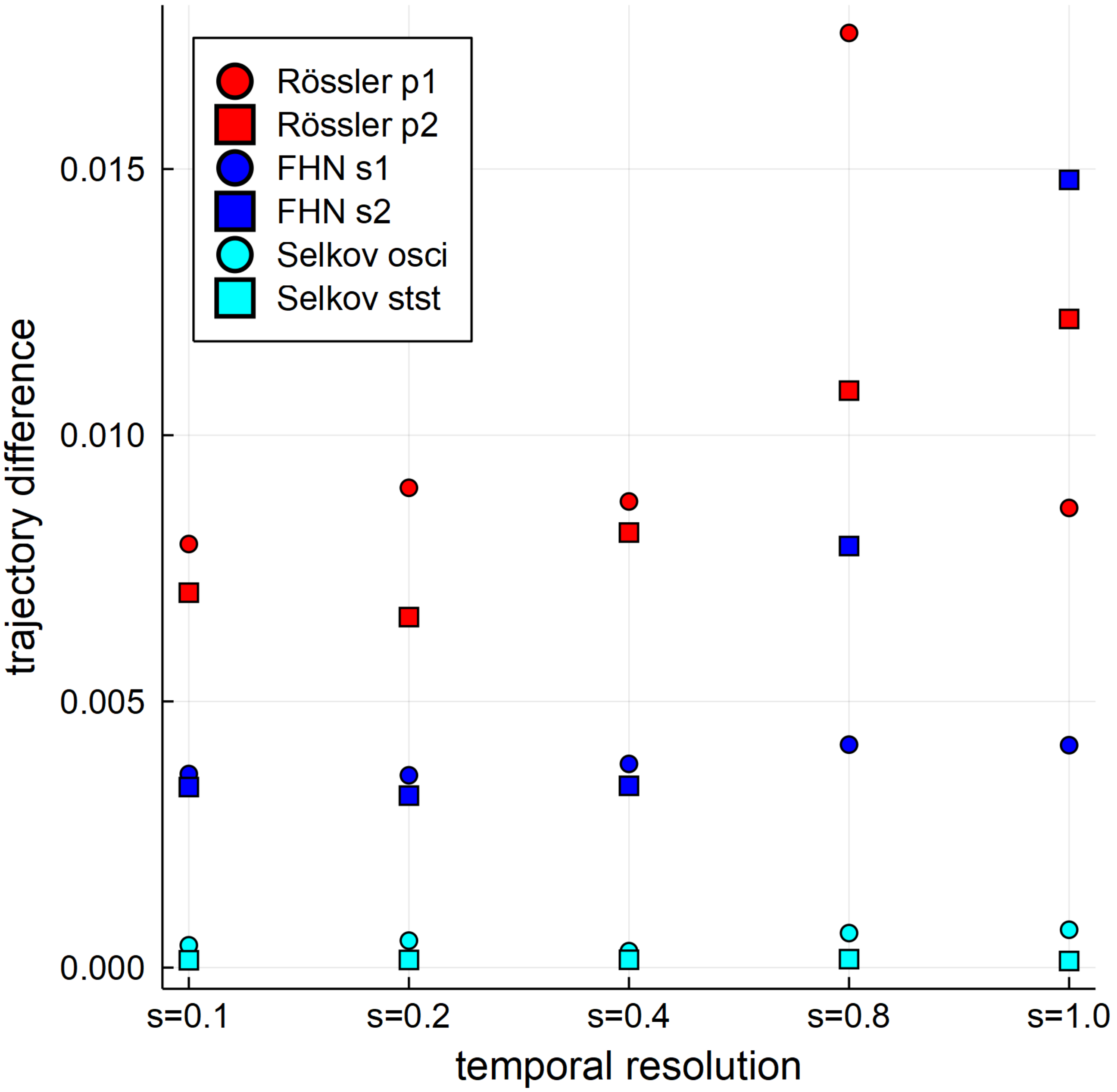

Figure S7. Impact of the training data's temporal resolution on the success of the approximation of the training data. One initial setting of the neural network was chosen and an example trajectory trained with LDA was estimated for both parametrizations of FitzHugh-Nagumo model (blue, monostable marked with circle, bistable with rectangle), Selkov model (cyan, oscillatory marked with circle, steady state with rectangle) (both models with noiselevel=0.0001) and Rössler model (red, period one marked with circle, period two with rectangle) (noiselevel=10^{-5}) for different temporal resolutions of the training data. As an independent measure the trajectory difference was calculated between the estimated solution and the training data and plotted against the temporal resolution.
This figure is plotted using Julia package Plots (Version v1.16.6, `https://github.com/JuliaPlots/Plots.jl`).

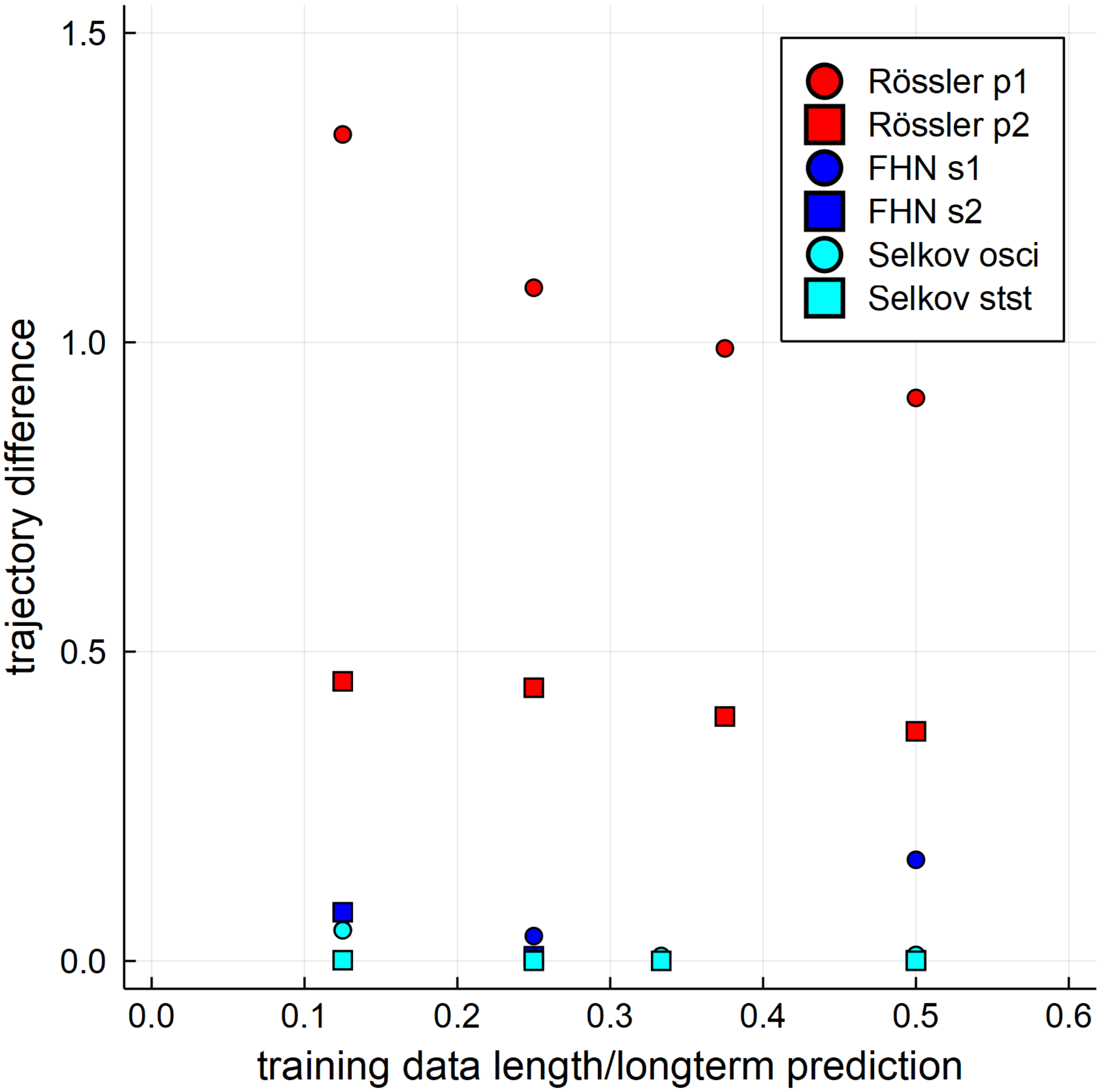

Figure S8. Impact of the training data length on the success of the approximation of the longterm prediction. One initial setting of the neural network was chosen and an example trajectory trained with LDA was estimated for both parametrizations of FitzHugh-Nagumo model (blue, monostable marked with circle, bistable with rectangle), Selkov meodel (cyan, oscillatory marked with circle, steady state with rectangle) (both models with noiselevel=0.0001) and Rössler model (red, period one marked with circle, period two with rectangle) (noiselevel=10^{-5}) for different training data legth. The longterm prediction timespan is fixed. As an independent measure the trajectory difference was calculated between the estimated longterm prediction and the true solution and plotted against the ratio of the training data length and the longterm prediction time span. This figure is plotted using Julia package Plots (Version v1.16.6, `https://github.com/JuliaPlots/Plots.jl`).
